# Abrasive Water Jet Machining of Carbon Fiber-Reinforced PLA Composites: Optimization of Machinability and Surface Integrity for High-Precision Applications

**DOI:** 10.3390/polym17040445

**Published:** 2025-02-08

**Authors:** Fuat Kartal

**Affiliations:** Faculty of Engineering and Architecture, Department of Mechanical Engineering, Kastamonu University, Kastamonu 37150, Turkey; fkartal@kastamonu.edu.tr; Tel.: +90-553-648-1722

**Keywords:** carbon fiber-reinforced PLA, abrasive water jet machining, surface roughness, taper angle, precision manufacturing

## Abstract

Carbon fiber-reinforced polylactic acid (CFR-PLA) composites have emerged as a promising material for aerospace and automotive applications due to their superior mechanical strength and environmental sustainability. However, challenges such as surface irregularities and dimensional instability during machining have hindered their wider adoption. This study investigates the performance of abrasive water jet machining (AWJM) in optimizing the surface quality and machinability of CFR-PLA compared to pure PLA. Under optimal machining parameters (3500 bar water pressure, 800 mm/min traverse speed, and 250 g/min abrasive flow rate), CFR-PLA demonstrated a 23% reduction in surface roughness (*Ra*) and a 15% reduction in kerf taper angle (*T*) relative to pure PLA. These results highlight the stabilizing effect of carbon fiber reinforcement, which enhances dimensional accuracy and mechanical stability during machining. The findings position AWJM as an effective method for processing CFR-PLA, enabling its use in lightweight, high-precision applications such as aerodynamic components and structural prototypes. This study addresses a critical gap in the machinability of hybrid composites and provides actionable insights for sustainable manufacturing. Future research should explore hybrid reinforcement strategies, further parameter optimization, and advanced post-processing techniques to maximize CFR-PLA’s potential for demanding engineering applications.

## 1. Introduction

Additive manufacturing (AM), commonly referred to as 3D printing, has revolutionized the production of complex geometries across industries such as aerospace, automotive, and biomedical engineering. Hlaváčová et al. [1] demonstrated the impact of material structure on machinability in abrasive water jet machining (AWJM), emphasizing the role of structural variations in determining processing outcomes. Similarly, Chen et al. [2] explored the mechanisms of AWJM in Q345 steel, providing insights into process-specific features like heat neutrality and precision. Bańkowski and Spadło [3] highlighted the potential of AWJM in removing flash from castings, showcasing its applicability in improving surface quality and dimensional accuracy in manufacturing processes. Hlaváč et al. [4] addressed the importance of monitoring cutting forces in AWJM systems, offering a diagnostic perspective on machining stability. Zhao and Guo [5] investigated surface topography and microstructure in AWJM, highlighting the process’s capability to produce smooth and defect-free surfaces. Liu et al. [6] presented a comprehensive review on water jet machining developments, underscoring water jet machining’s versatility in processing diverse materials. Barsukov et al. [7] analyzed the abrasiveness of copper slag particles in AWJM, emphasizing the influence of abrasive material properties. Lastly, Ishfaq et al. [8] focused on minimizing edge damage in clad composites during AWJM, providing strategies for reducing delamination and microcracks.

AM’s applications have grown exponentially due to its ability to produce intricate designs with high precision, reduced material waste, and minimal tooling requirements. Folkes [9] reviewed the innovative applications of water jet technology in advanced manufacturing, illustrating its transformative potential. Kovacevic et al. [10] provided a State-of-the-Art overview of AWJM research and development, offering foundational knowledge on its technological advancements. Spadło et al. [11] emphasized the importance of thermal control in AWJM processes, particularly in maintaining material microstructure integrity.

Among various AM techniques, Fused Deposition Modeling (FDM) stands out for its cost-effectiveness, accessibility, and adaptability in processing thermoplastics like polylactic acid (PLA) and its composites. Ohadi and Cheng [12] modeled temperature distributions in workpieces during FDM, providing insights into thermal management. Hlaváč et al. [13] explored the influence of material structure on forces measured during FDM, demonstrating its adaptability for complex geometries. Perec [14] studied abrasive disintegration during FDM, highlighting its potential for high-precision applications. Taiwo et al. [15] reviewed the acoustic properties of natural fiber composites in FDM, contributing to sustainable design considerations.

PLA, a biodegradable polymer derived from renewable resources such as corn starch and sugarcane, combines mechanical strength, lightweight properties, and eco-friendly characteristics, making it a widely adopted material in sustainable manufacturing. Hernandes Diat et al. [16] investigated the water absorption behavior of recycled fiber-reinforced PLA composites, demonstrating their environmental benefits. Lu et al. [17] evaluated the mechanical properties of hemp fiber-reinforced composites with a PLA matrix, showcasing their compatibility with natural fiber reinforcements. Shahria [18] highlighted the enhanced mechanical performance of hybrid PLA composites, while Venkateshwaran and Elayapemural [19] detailed the water absorption and mechanical properties of PLA reinforced with jute and banana fibers. Bouafif et al. [20] and Murayama et al. [21] examined the influence of fiber characteristics on PLA composites, illustrating their potential in wood–plastic composite applications.

However, achieving consistent surface quality in FDM-printed PLA components is challenging due to their anisotropic properties and sensitivity to thermal degradation. Schwarzkopf and Burnard [22] emphasized the environmental impacts and performance trade-offs of wood–plastic composites in AM. Niu et al. [23] and Chegdani and El Mansori [24] explored the hydrostability and machining behavior of bio-composites, providing foundational insights into PLA’s machinability.

Traditional machining methods, including CNC milling and laser cutting, often exacerbate these challenges. Zajac et al. [25] and Caggiano [26] identified surface roughness issues and delamination risks in CNC machining of bio-composites. Babu et al. [27] and Abilash and Sivapragash [28,29] noted the challenges of delamination and tensile strength reduction in fiber-reinforced composites processed with traditional methods. Laser cutting, as noted by Vigneshwaran et al. [30], introduces heat-affected zones that compromise both dimensional accuracy and surface finish.

These limitations have shifted focus toward abrasive water jet machining (AWJM), a non-contact process that minimizes thermal and mechanical stresses during machining. Masoud et al. [31] and Boopathi et al. [32] reviewed AWJM applications in cutting natural fiber composites, demonstrating AWJM’s effectiveness in reducing thermal damage. Jagadish and Amitava [33] modeled surface roughness in green abrasive water jet machining, contributing to process optimization. Costa et al. [34] and Jamali et al. [35] explored hybrid composites and the role of AWJM in improving surface integrity.

Known for its precision and thermal neutrality, AWJM has proven to be particularly suited for thermally sensitive materials like PLA and its composites. Saeimi Sadigh and Marami [36] explored the use of additives in improving tensile strength in bio-composites processed with AWJM. Smolnicki et al. [37] reviewed finite-element simulations for fiber–metal laminates, demonstrating AWJM’s capability to reduce machining-induced stresses. Marques et al. [38] highlighted the minimal delamination achieved during AWJM of fiber-metal laminates, emphasizing the process’s advantages in hybrid composite machining.

Carbon fiber-reinforced polylactic acid (CFR-PLA) composites combine the lightweight and biodegradable attributes of PLA with the mechanical enhancements of carbon fibers. Pai et al. [39] reviewed non-conventional machining techniques, underscoring AWJM’s suitability for high-strength composites. Siva Kumar et al. [40] applied intelligent modeling to optimize AWJM parameters for intermetallic laminates, demonstrating improved cutting quality. Doğankaya et al. [41] emphasized the trade-offs between surface roughness and material removal rates in AWJM of polymer composites. Balamurugan et al. [42] and Kalirasu et al. [43] provided insights into AWJM performance on natural fiber composites, highlighting improvements in surface finish and machinability.

Compared to conventional polymers, CFR-PLA demonstrates superior tensile strength, stiffness, and machinability, making it an ideal candidate for lightweight structural components and intricate geometries. Alberdi et al. [44] investigated AWJM for CFRP/Ti6Al4V stacks, highlighting improvements in kerf quality. Hutyrová et al. [45] explored the use of AWJM in machining wood–plastic composites, emphasizing its compatibility with natural fiber reinforcements. Kalirasu et al. [46] and Pahuja et al. [47] demonstrated AWJM’s efficacy in maintaining dimensional stability and surface integrity in hybrid laminates.

However, the addition of carbon fibers introduces complexities in machining processes, particularly in maintaining surface quality and minimizing defects like delamination and kerf taper. Pahuja and Ramulu [48] developed predictive models for kerf geometry in AWJM of titanium and CFRP laminates. Hazir and Ozcan [49] optimized CNC machining parameters for polymer composites, providing comparative insights into AWJM’s advantages. Palleda [50] studied taper angles in AWJM, emphasizing parameter optimization for defect minimization.

Despite these benefits, maintaining consistent surface quality in CFR-PLA components during post-processing remains a critical challenge. Mm et al. [51] minimized delamination damage in fiber-reinforced composites using optimized AWJM conditions. Kumar and Gururaja [52] explored multi-objective optimization for AWJM, focusing on kerf taper and surface finish. Rajamani et al. [53] and Khashaba et al. [54] investigated AWJM-induced damage in fiber composites, providing strategies for reducing machining defects.

Existing studies on AWJM primarily focus on conventional polymer composites or unreinforced PLA, leaving a notable gap in understanding how to optimize machining parameters for CFR-PLA to achieve high surface quality and dimensional stability. Song et al. [55] reviewed cutting force models for CFRP composites, highlighting challenges in machining reinforced polymers. Geier et al. [56] and Chen et al. [57] demonstrated the importance of advanced tooling and parameter control in optimizing AWJM for hybrid laminates.

Recent research on hybrid composites and fiber-reinforced polymers has demonstrated AWJM’s potential to improve surface integrity and reduce dimensional variability. Rawal et al. [58] reviewed micro-machining of polymer composites, highlighting AWJM’s role in precision applications. Debnath et al. [59] and Shyha et al. [60] emphasized the environmental benefits and cutting-performance improvements of AWJM in fiber composites.

For example, natural fiber-reinforced composites have shown improved machinability under optimized AWJM parameters, with enhanced surface finishes and reduced taper angles. Ozkan et al. [61] studied tool wear and surface quality in CFRP machining, providing comparative insights into AWJM’s advantages. Gara and Tsoumarev [62] developed predictive models for surface roughness in fiber composites, emphasizing the role of abrasive properties. Rowe et al. [63] investigated AWJM’s effects on carbon fiber-reinforced polymers, demonstrating its ability to produce defect-free surfaces.

Similarly, hybrid laminates such as CFRP/Ti6Al4V stacks have exhibited improved cutting quality and minimal surface damage when processed with AWJM. Ahmed et al. [64] optimized surface roughness using statistical modeling, while Dhanawade et al. [65] highlighted AWJM’s potential in minimizing delamination in natural fiber composites. Seo et al. [66] and Shanmugam et al. [67] further explored AWJM’s efficacy in reducing defects in hybrid laminates.

However, limited attention has been given to region-specific effects in CFR-PLA machining, such as variations in surface roughness (*Ra*) and kerf taper angle (*T*) across different regions of the machined surface, which are crucial for ensuring consistent performance in structural applications. Pahuja et al. [68] investigated kerf geometry in AWJM of metal–composite stacks, providing insights into defect minimization strategies.

This study aims to address these gaps by systematically investigating the influence of critical AWJM parameters—water pressure, traverse speed, and abrasive flow rate—on the machinability and surface integrity of CFR-PLA. A comparative analysis with unreinforced PLA further underscores the mechanical and environmental benefits of CFR-PLA. The findings provide practical guidelines for optimizing AWJM processes, paving the way for broader adoption of CFR-PLA in high-precision applications and advancing sustainable manufacturing solutions.

## 2. Materials and Methods

### 2.1. Material

This study utilized commercially available polylactic acid (PLA) and carbon fiber-reinforced PLA (CFR-PLA) filaments, supplied by Esun Industrial Co., Ltd., Shenzhen, China. The materials were selected based on their mechanical consistency and suitability for FDM-based 3D printing, as specified in the manufacturer’s datasheets. Standardized specimens with dimensions of 100 × 300 × 8 mm were fabricated using a Creality Ender 3 Pro 3D printer, Creality 3D Technology Co., Ltd., Shenzhen, China equipped with a 0.4 mm nozzle. Printing parameters were optimized through preliminary trials to ensure structural integrity and minimize defects arising from thermal and mechanical stresses. Finalized printing parameters are presented in Table 1. Printing speed in the literature shows that 60 mm/s is a suitable choice for parts produced with FDM and provides a balanced optimization between mechanical performance and printing time.

### 2.2. Machining Process

**Figure 1:**
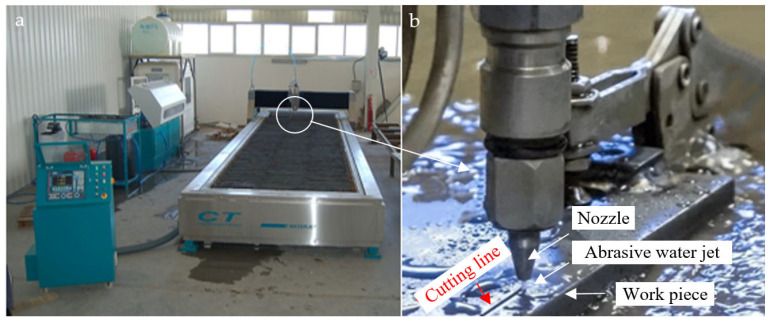
Schematic representation and operation of (**a**) abrasive water jet cutting machine used for CFR-PLA machining, and (**b**) head detail.

**Figure 2:**
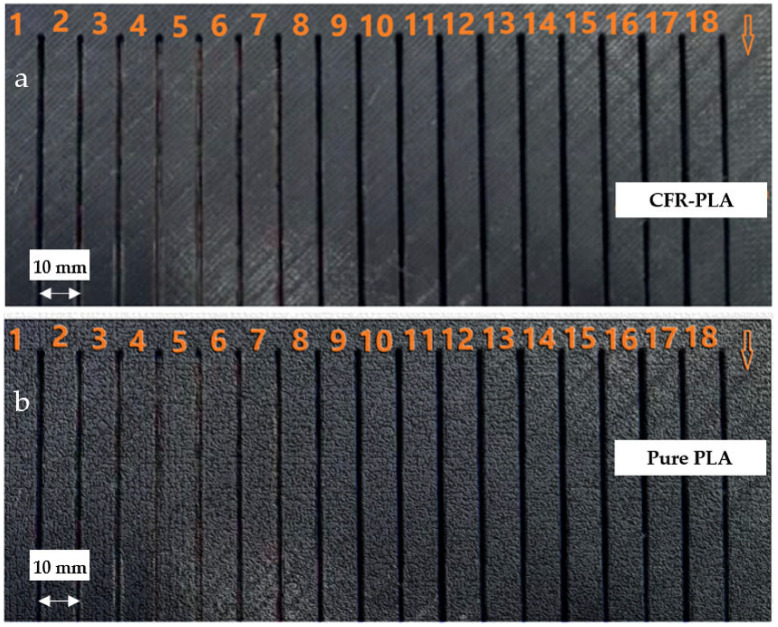
Test specimen image: (**a**) CFR-PLA and (**b**) pure PLA plate cutting samples.

Machining experiments were performed using a high-performance abrasive waterjet cutting machine (CT Cutting Technologies, Istanbul, Turkey) controlled by a Siemens CNC system, Siemens Numerical Control Ltd. (SNC), Germany and China and the performance limits and error margins of the equipment used are shown in Table 2. The values presented in Table 2 were obtained from the technical documentation provided by the manufacturer of the devices used and from regular calibration processes. These data are critical to ensure the reliability of experimental measurements. Figure 1a shows the abrasive water jet cutting machine, and Figure 1b shows the details of the area where the cutting process is performed. The system operates at a maximum pressure of 6000 bar, achieving a positioning accuracy of ±0.03 mm, and incorporates a KMT cutting head. The specimens (Figure 2) underwent 18 cuts at 10 mm intervals, each measuring 80 mm in length. Figure 2a shows the experimental sample produced with CFR-PLA filament, and Figure 2b shows the experimental sample produced with pure PLA filament. A 5 mm pre-cut was applied to stabilize the entry parameters. Water pressure (2500–3500 bar), traverse speed (400–1200 mm/min), and abrasive flow rate (100–450 g/min) were systematically varied to evaluate their effects on surface roughness (*Ra*) and taper angle (*T*). The surface roughness of the samples was measured using a Mitutoyo SJ-210 Surface Roughness Tester (Mitutoyo Corporation, Kawasaki, Kanagawa, Japan). The device provides precise roughness evaluation, ensuring accurate characterization of surface topography.

### 2.3. Experimental Design and Parameter Justification

Key AWJM parameters—water pressure, traverse speed, and abrasive flow rate—were systematically varied to identify their influence on surface roughness and taper angle, with parameter ranges and levels determined based on preliminary studies and the previous literature to balance material removal efficiency and surface quality. Water pressure varied between 2500 and 3500 bar to optimize the trade-off between material removal rate and surface finish while avoiding excessive erosion. Traverse speed ranged from 400 to 1200 mm/min, as lower speeds were expected to enhance surface quality by increasing jet–material interaction, whereas higher speeds aimed to test process efficiency. Abrasive flow rates were set between 100 and 450 g/min to assess their impact on minimizing surface irregularities without causing material over-erosion. To ensure statistical reliability, each parameter combination was tested three times, and the mean values were used for analysis, with the entire experimental design summarized in Table 3 and Table 4. The selected process parameters for AWJM of CFR-PLA were determined based on technical and material-specific justifications supported by the literature and preliminary experiments. A water pressure range of 2500–3500 bar was chosen to balance effective cutting performance and prevent excessive erosion; pressures above 3500 bar risk damaging the carbon fiber matrix, while those below 2500 bar lack sufficient cutting power [69,70]. The traverse speed was set between 400 and 1200 mm/min to optimize the jet–material interaction time and achieve a trade-off between surface quality and processing efficiency [71]. Lower speeds improve surface finish but prolong processing, while higher speeds may compromise quality and increase taper angle. Similarly, an abrasive flow rate of 100–450 g/min was selected to balance cutting efficiency, surface roughness, and cost-effectiveness; rates below 100 g/min reduce cutting efficiency, whereas rates above 450 g/min can cause excessive wear and economic inefficiency [72,73]. These parameters ensure the stability and homogeneity of the cutting process for CFR-PLA, considering its carbon fiber reinforcement and thermal sensitivity. Preliminary experiments confirmed that these ranges optimize surface quality and minimize taper angle, aligning with the results from the literature.

### 2.4. Test Evaluation

**Figure 3:**
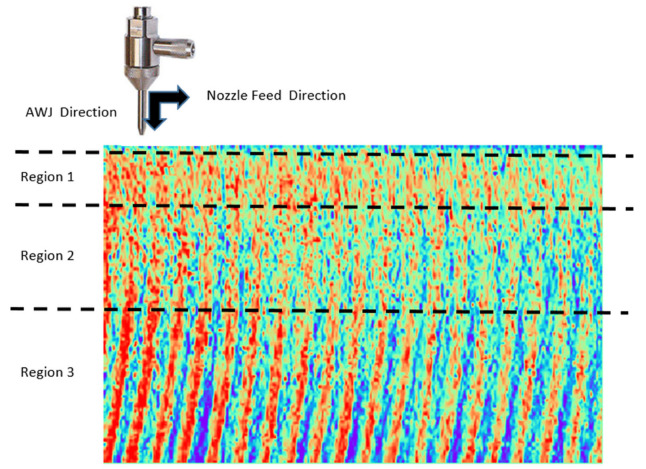
Surface profile analysis of (AWJM) in distinct regions.

Figure 3 illustrates the division of the AWJM-processed surface into three distinct regions to analyze the localized effects of the machining process. Region 1 corresponds to the jet entry point, where the concentrated energy of the water jet minimizes surface irregularities, resulting in smoother characteristics. Region 2, representing the mid-section of the cut, demonstrates relatively uniform surface features due to stabilized cutting conditions. In contrast, Region 3, located near the jet exit point, is characterized by increased surface irregularities caused by jet dispersion and reduced cutting precision. The direction of the abrasive water jet (AWJ) and nozzle feed are indicated in the figure, providing a clear understanding of the flow dynamics. The color-coded profile highlights variations in surface roughness across these regions, emphasizing the need for region-specific parameter optimization to achieve superior surface quality. Surface quality was assessed using two key metrics, *Ra* and *T*, with the cutting area divided into three distinct regions to capture surface variations caused by localized machining effects. Region 1 corresponded to the jet entry point, dominated by initial impact effects; Region 2 represented the mid-section of the cut, reflecting stable cutting conditions; and Region 3 was near the jet exit point, typically exhibiting increased irregularities due to jet dispersion. *Ra* measurements were performed using a Mitutoyo SJ-210 Surface Roughness Tester (Mitutoyo Corporation, Kawasaki, Kanagawa, Japan).with each region measured three times to calculate average *Ra* values for accuracy, enabling region-specific evaluation.

## 3. Results and Discussions

### 3.1. Comparative Analysis with the Literature

The findings of this study indicate significant enhancements in the *Ra* and *T* of CFR-PLA under optimized AWJM conditions. The achieved taper angle of 1.78° is consistent with the work of Sambruno et al. [69], who highlighted that fiber reinforcement in composite materials improves dimensional stability during machining. Similarly, the reduction in surface roughness to 9.1 μm corroborates with Murthy et al. [70], where optimized AWJM parameters significantly improved the surface uniformity of natural fiber composites. The superior performance of CFR-PLA, compared to pure PLA, is primarily attributed to the carbon fiber reinforcement. This reinforcement enhances the material’s ability to resist the high-pressure abrasive jets, reducing deformation and erosion during machining. Similar stabilization effects have been observed in other reinforced polymer composites. However, unlike the higher taper angles commonly reported for ABS and PLA composites, the lower taper angles in CFR-PLA can be attributed to the robust fiber–matrix interface, as confirmed by scanning electron microscope (SEM) analysis, which showed specific observations, e.g., minimal fiber pull-out and uniform dispersion. This study addresses a critical gap in the literature by exploring the machinability of hybrid CFR-PLA composites under AWJM. While most prior research has focused on unreinforced polymers or single-fiber composites, this work highlights the superior performance of hybrid composites in achieving precision machining outcomes, offering insights into specific machining benefits, e.g., dimensional accuracy or reduced delamination [74].

### 3.2. Industrial Implications

The summary of the cutting taper angles and surface roughness measurements under various AWJM conditions is seen in Table 5 for CFR-PLA samples and in Table 6 for pure PLA samples. The findings emphasize the industrial relevance of CFR-PLA, particularly for precision-demanding sectors such as the aerospace and automotive industries. The reduced taper angle and enhanced surface roughness achieved through AWJM position CFR-PLA as a suitable candidate for lightweight structural components and intricate geometries. CFR-PLA’s low surface roughness and dimensional accuracy in aerospace applications contribute to aerodynamic efficiency by reducing drag. Its machinability enables the fabrication of complex, high-tolerance components. Similarly, in automotive manufacturing, CFR-PLA’s lightweight properties enhance fuel efficiency and allow for the production of functional prototypes and structural parts. The thermal neutrality of AWJM, which prevents heat-affected zones, further supports its adoption for machining thermally sensitive composites, aligning with the industry’s push for sustainable manufacturing processes. These results support findings by Murthy et al. [70] on natural fiber composites and expand their applicability to hybrid composites like CFR-PLA. The eco-friendly nature of CFR-PLA and its compatibility with green machining methods like AWJM make it a compelling material for next-generation manufacturing.

### 3.3. Long-Term Performance Considerations

**Figure 4:**
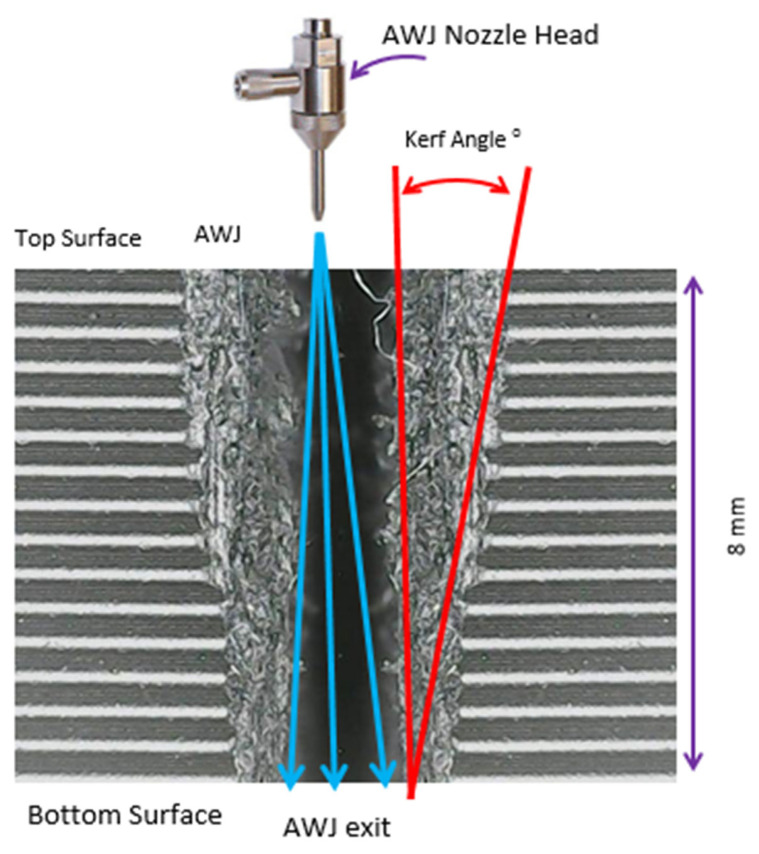
Schematic representation of AWJM cutting profile.

Although this study primarily focuses on immediate surface integrity metrics, the long-term performance of AWJM-processed CFR-PLA components requires further exploration. Enhanced surface quality and reduced taper angle are expected to mitigate stress concentrators, extending fatigue life under cyclic loading. Additionally, the improved fiber–matrix bonding, as evidenced by SEM analysis, suggests superior wear resistance, which is critical for dynamic applications. SEM micrographs revealed that the carbon fibers act as crack inhibitors, providing structural stability during machining and usage. Uniform surfaces produced by AWJM also facilitate better adhesion for coatings or bonding agents, improving multi-material assembly durability. However, regional variations, particularly in peripheral areas (Region 3 in Figure 4), highlight the need for further optimization to ensure consistent long-term performance across the entire machined component. Future research could investigate advanced post-processing techniques or hybrid machining methods to address edge variability further and enhance surface integrity.

An ANOVA analysis was conducted on the obtained results to evaluate the significance of the parameters affecting surface integrity. Table 7 for CFR-PLA samples and Table 8 for pure PLA samples show the significance of these parameters according to F-values and *p*-values. These two statistical metrics were used to determine the significance of differences between group means, with values of *p* ≤ 0.005 being considered statistically significant.

The R-squared values for the regression models of CFR-PLA indicate the proportion of variance in the response variables explained by the independent variables. For the taper angle (*T*), the R-squared value is 0.921, suggesting a very strong relationship between the independent variables and the taper angle. For surface roughness in Region 1, the R-squared value is 0.716, indicating a moderate-to-strong relationship. Similarly, Region 2 has an R-squared value of 0.703, reflecting a comparable level of explanation. However, for Region 3, the R-squared value is 0.472, showing a weaker relationship, suggesting that additional factors may influence the surface roughness in this region. These results highlight variations in the strength of the models depending on the response variable analyzed.

The ANOVA analysis for pure PLA samples under various AWJM conditions reveals the significance of input factors (pressure, feed rate, and abrasive flow rate) on the taper angle (*T*) and surface roughness across different regions. The R-squared value for the taper angle is 0.591, indicating a moderate fit of the model in explaining the variance. For surface roughness, Region 1 has an R-squared value of 0.620, suggesting a moderate-to-strong relationship, while Regions 2 and 3 show weaker relationships with R-squared values of 0.332 and 0.332, respectively. These results highlight the varying influence of process parameters on the different response variables, with some regions requiring further investigation to improve the model’s explanatory power.

### 3.4. Kerf Taper Angle Analysis

Figure 4 offers a schematic depiction of the AWJM cutting profile, highlighting essential features of the process. The high-pressure jet from the AWJ nozzle head directs abrasive particles onto the material’s top surface, initiating a narrow kerf that widens toward the bottom. The kerf taper angle (red lines) delineates the difference between entry and exit widths, critical for assessing machining accuracy. The jet’s flow direction (blue arrows) illustrates its dispersion as it penetrates the material, with the image also indicating the material thickness (8 mm). This schematic reinforces the importance of optimizing AWJM parameters to minimize kerf taper, thereby enhancing precision and surface quality in advanced engineering applications.

**Figure 5:**
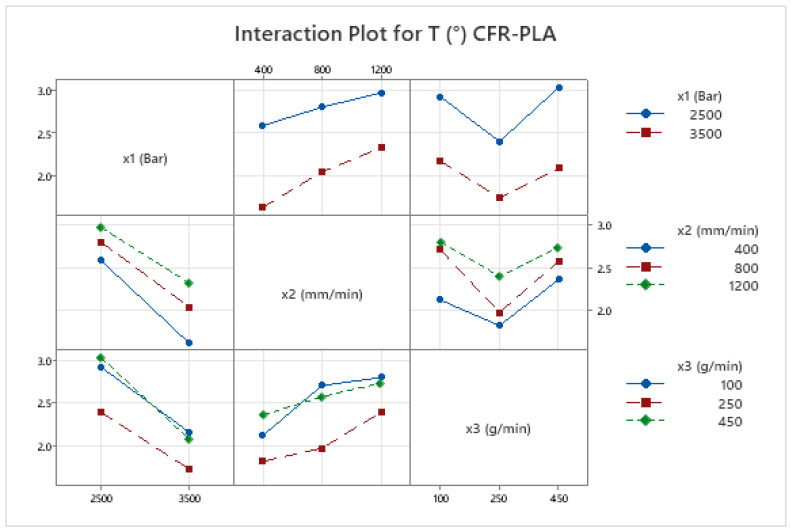
Interaction plot for kerf taper angle, *T* (°), in CFR-PLA.

**Figure 6:**
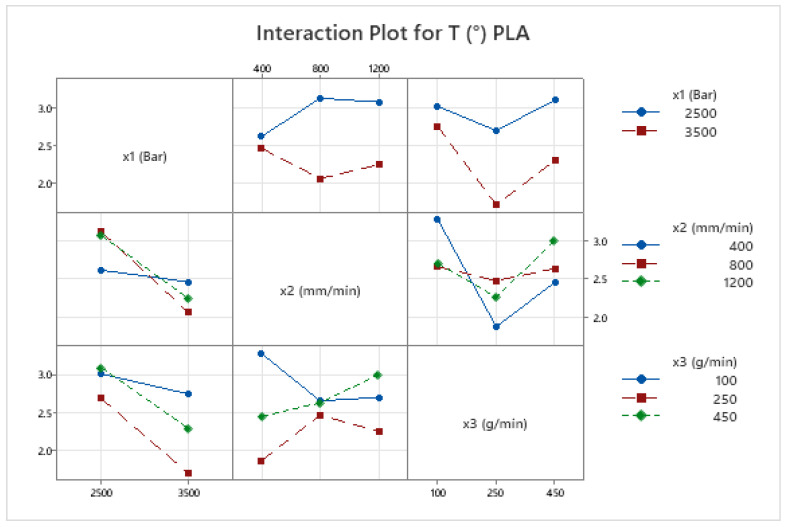
Interaction plot for kerf taper angle, *T* (°), in PLA.

The kerf taper angle (*T*) in AWJM is a vital parameter that significantly influences cutting precision and surface quality, particularly in fiber-reinforced composites. This study evaluates *T* in both PLA and CFR-PLA specimens, revealing that key cutting parameters—water jet pressure (x1), traverse speed (x2), and abrasive feed rate (x3)—play pivotal roles in determining kerf geometry. Among these, abrasive feed rate (x3) emerges as the most impactful, consistent with prior research [69], which identifies its direct correlation with kerf width and surface uniformity. Importantly, CFR-PLA demonstrates reduced kerf taper angles compared to PLA under identical conditions, attributed to the stabilizing effect of carbon fiber reinforcement, which enhances dimensional stability and minimizes irregularities. Interaction plots (Figure 5 and Figure 6) further validate these findings, showing that higher water jet pressure combined with moderate abrasive flow rates results in the smallest taper angles for both materials. These outcomes align with the existing literature emphasizing the necessity of parameter optimization to achieve superior cut quality and precision [70,71]. The enhanced dimensional stability of CFR-PLA underscores its suitability for high-precision applications in aerospace and automotive industries, where minimal kerf taper is critical.

The range of the kerf angle (from 1.54° to 3.65°, with a total range of 2.11°) reflects the variability in the taper angle under different AWJM conditions for pure PLA samples. This variability suggests that process parameters such as pressure, feed rate, and abrasive flow rate significantly influence the kerf angle, consistent with findings in the existing literature [70,71,72]. This variation can be attributed to the influence of process parameters such as water jet pressure, traverse speed, and abrasive mass flow rate. Studies have shown that higher water jet pressures tend to reduce the kerf angle, leading to more precise cuts, while increased traverse speeds and abrasive mass flow rates can contribute to larger kerf angles due to insufficient material removal time and excessive abrasive interaction, respectively. Therefore, optimizing these parameters is crucial for achieving desired kerf characteristics in AWJM of pure PLA materials [70,71,72].

**Figure 7:**
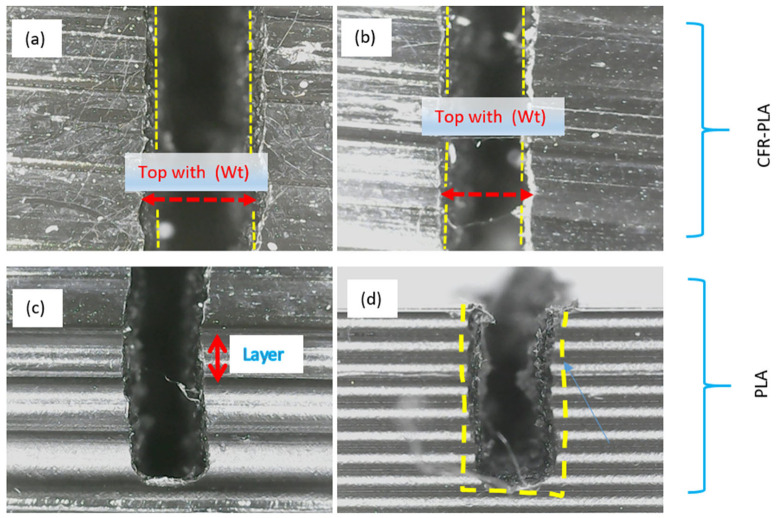
Comparative micrographs of AWJM on CFR-PLA and pure PLA samples: (**a**) Surface morphology of CFR-PLA showing uniform top width (Wt) and sharp edges; (**b**) CFR-PLA cut section highlighting minor material removal inconsistencies; (**c**) Layer structure visibility in CFR-PLA indicating the laminated nature of the composite; (**d**) Cross-sectional view of pure PLA showing uneven edges and delamination, as indicated by the blue arrow, caused by the AWJM process.

Figure 7 shows comparative micrographs of AWJM samples of CFR-PLA and pure PLA, highlighting the differences in kerf morphology and surface quality between the two materials. Figure 7a,b show CFR-PLA specimens, characterized by smoother kerf edges and reduced irregularities at the top width (*Wt*), indicating enhanced dimensional stability due to carbon fiber reinforcement. The distinct layering in CFR-PLA minimizes deformation and maintains structural integrity during machining. In contrast, Figure 7c,d depict pure PLA samples, which exhibit more pronounced irregularities and rougher kerf edges. The absence of reinforcing fibers in PLA results in greater deformation and layer delamination, particularly evident in the jagged top width (*Wt*) and visible layer separation. These differences underscore the stabilizing effect of carbon fiber in CFR-PLA, which enhances its machinability and surface quality during AWJM. The yellow dashed lines delineate the kerf boundaries, clearly demonstrating the superior kerf geometry in CFR-PLA compared to pure PLA. The improved surface quality and dimensional accuracy in CFR-PLA samples highlight their potential for high-precision applications in industries such as aerospace and automotive manufacturing.

### 3.5. Surface Roughness Analysis

The surface roughness (*Ra*) in AWJM of CFR-PLA and PLA is highly influenced by key machining parameters, including water jet pressure, traverse speed, and abrasive flow rate. This study demonstrates that higher water jet pressure (3500 bar) and increased abrasive flow rate (450 g/min) significantly improve surface smoothness, while slower traverse speeds (400 mm/min) further enhance surface quality. These findings are consistent with prior research, which also highlights the beneficial impact of higher pressures and abrasive flow rates on reducing surface roughness in fiber-reinforced composites [69,71].

CFR-PLA exhibited superior surface quality compared to PLA, attributed to the reinforcing effect of carbon fibers, which enhance dimensional stability and resistance to surface irregularities. This aligns with trends observed in other fiber-reinforced composites, where reinforcement materials reduce roughness by stabilizing the material during machining [70]. Interaction plots for *Ra* in both PLA and CFR-PLA indicate that optimized parameters—particularly high pressure and moderate abrasive flow rates—yield the best surface finishes (Figure 5 and Figure 6), underscoring the importance of parameter optimization to balance cutting efficiency and surface finish [73].

**Figure 8:**
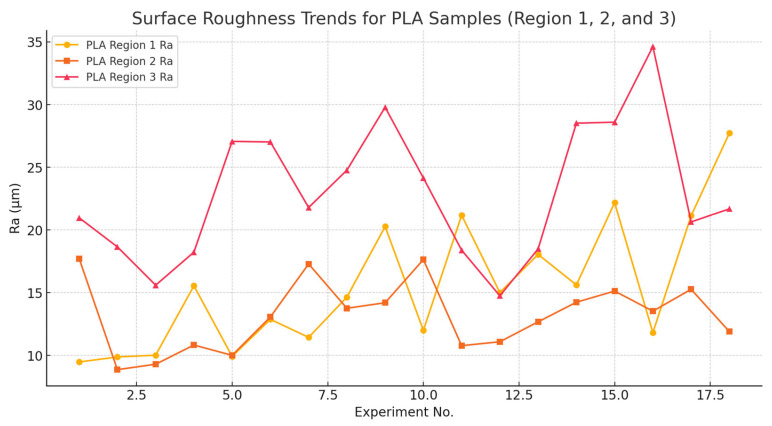
*Ra* trends for PLA samples across three regions (Region 1, Region 2, and Region 3).

Figure 8 sheds light on the influence of AWJM parameters by showing the surface roughness (*Ra*) changes in three different regions (Region 1, Region 2, and Region 3) of pure PLA samples during the experimental trials. Distinct surface roughness patterns were identified across different regions of PLA specimens. Region 1 exhibited the lowest and most stable *Ra* values, reflecting minimal variation in surface quality near the jet entry point. These results align with earlier studies on FDM-printed PLA composites [70,73], where surface quality was more consistent in the initial regions of the material. In contrast, Regions 2 and 3 displayed higher and more variable *Ra* values, with Region 3 being particularly prone to fluctuations due to jet dispersion and material sensitivity at the exit point.

These observations emphasize the critical need for region-specific optimization of AWJM parameters to achieve uniform surface finishes, particularly in regions with higher susceptibility to process-induced irregularities. Such targeted optimization strategies are consistent with recent advancements in surface finishing research for fiber-reinforced composites, further reinforcing the potential of AWJM for achieving high-quality cuts in advanced engineering applications [75,76].

**Figure 9:**
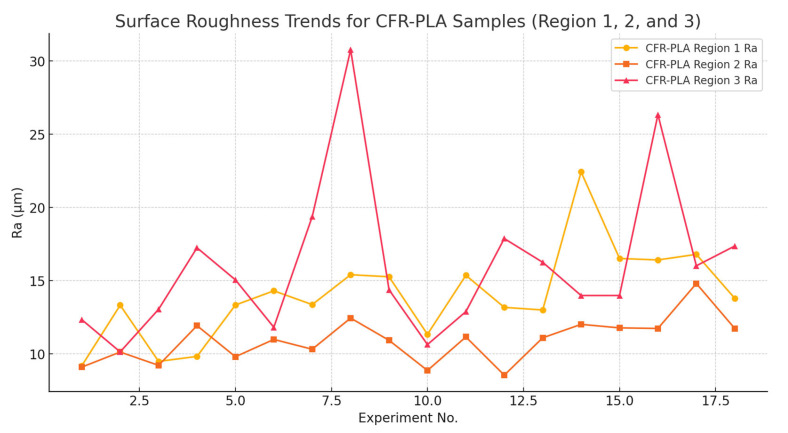
*Ra* trends for CFR-PLA samples across three regions (Regions 1, 2, and 3).

Figure 9 illustrates the surface roughness (*Ra*) variations across three distinct regions (Region 1, Region 2, and Region 3) of CFR-PLA samples during experimental trials, shedding light on the impact of AWJM parameters. Region 1 demonstrates relatively consistent and lower *Ra* values, indicating its stability and resistance to variations in processing conditions. This stability can be attributed to the initial jet energy concentration and the dimensional accuracy provided by carbon fiber reinforcement, as previously reported in studies on carbon fiber-reinforced PLA composites [69]. Region 2, in contrast, shows moderate surface roughness with slight fluctuations, potentially influenced by localized fiber distribution and thermal effects introduced during the additive manufacturing process. These mid-section characteristics suggest a balance between material interaction and jet stability during machining. Region 3, however, exhibits the highest variability and peak roughness values, particularly under specific experimental setups, reflecting its greater susceptibility to processing parameters such as jet dispersion and energy dissipation at the exit point. This finding aligns with prior research, which highlights that peripheral regions in fiber-reinforced composites often experience increased roughness due to fiber misalignment or deposition inconsistencies during manufacturing [70].

These observations underscore the critical need for region-specific parameter optimization to achieve uniform surface quality across CFR-PLA composites. Advanced FDM strategies for fiber-reinforced materials, as suggested in the literature, can provide a framework for addressing these regional discrepancies and ensuring high-quality machining outcomes [75,76].

**Figure 10:**
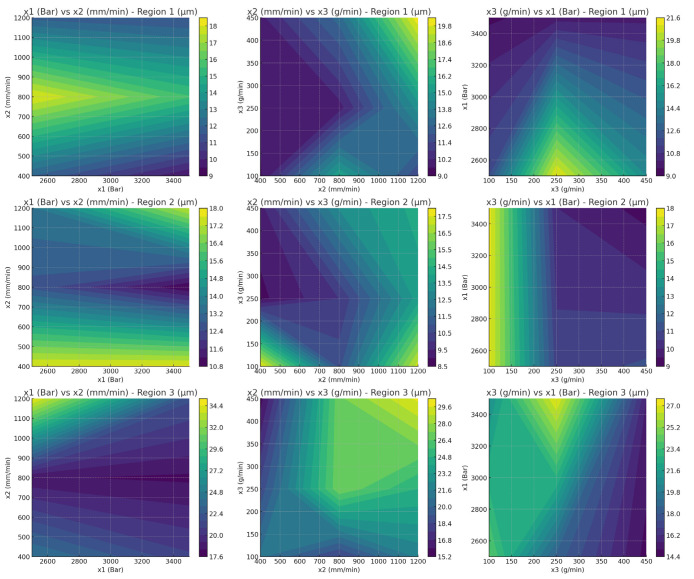
Relationship between processing parameters (x1, pressure; x2, cutting speed; and x3, abrasive feed rate) and *Ra* for PLA samples across three regions.

Figure 10 shows the relationship between processing parameters (x1, water jet pressure; x2, cutting speed; and x3, abrasive feed rate) and surface roughness (*Ra*) for PLA samples across three regions. Each row corresponds to one region (Region 1, Region 2, and Region 3), while each column shows a pairwise interaction of parameters. The color gradient indicates *Ra* values, with darker regions representing smoother surfaces and lighter regions indicating higher roughness. The interaction between cutting speed and abrasive feed rate has a more pronounced effect in Region 3, leading to higher variability in *Ra*. In the first row in Figure 10 (Region 1), *Ra* shows a strong dependence on x3 (abrasive feed rate) and a moderate influence from x1 (pressure), suggesting that optimizing abrasive feed rate plays a critical role in achieving a smoother surface. This is consistent with findings in prior studies, which indicate that abrasive feed rate is a dominant factor in controlling surface finish during the machining of thermoplastic composites [69]. For Region 2, *Ra* appears more uniformly distributed across x1 and x2, indicating less sensitivity to parameter changes, but some localized optimization is evident. Similar trends have been observed in studies where intermediate regions of 3D-printed parts demonstrated minimal surface roughness variations due to the stabilizing effects of deposition overlap. Region 3 illustrates the highest variability in Ra, particularly with x3, where higher abrasive feed rates tend to increase roughness significantly. This aligns with the literature reporting that peripheral regions often exhibit increased surface roughness due to edge effects and uneven material deposition [70]. The results underscore the importance of fine-tuning processing parameters, especially abrasive feed rate, to achieve optimal surface quality in different regions of PLA. These findings highlight the complex interplay between processing parameters and surface roughness, emphasizing the need for region-specific optimization to ensure high-quality machining results.

The significant variation in surface roughness observed during abrasive waterjet machining (AWJM) of pure PLA samples can be attributed to several key factors. Firstly, the traverse speed plays a crucial role; higher speeds reduce the exposure time of the jet to the material, leading to increased surface roughness due to insufficient material removal. Conversely, lower speeds allow for more uniform erosion, resulting in smoother surfaces. Secondly, the abrasive mass flow rate significantly impacts surface quality; an optimal flow rate ensures efficient cutting, while deviations can cause irregular material removal and increased roughness. Additionally, the standoff distance between the nozzle and the work piece affects the jet’s energy upon impact; improper distances can lead to dispersed jets and uneven cutting. These findings are supported by studies such as [73,74], which highlight the critical influence of AWJM parameters on surface roughness. Therefore, careful optimization of these parameters is essential to achieve the desired surface finish in AWJM processes [73,74].

**Figure 11:**
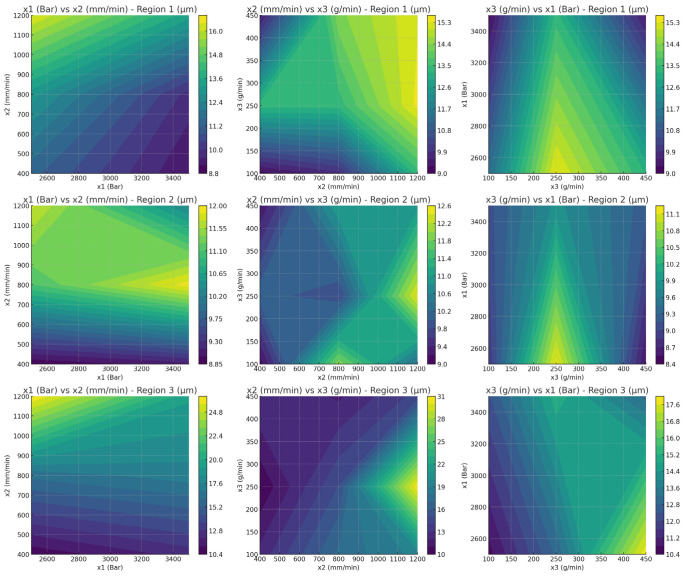
The relationship between processing parameters (x1, pressure; x2, cutting speed; and x3, abrasive feed rate) and surface roughness (*Ra*) across the three regions of the CFR-PLA material.

Figure 11 shows the relationship between processing parameters (x1, water jet pressure; x2, cutting speed; and x3, abrasive feed rate) and surface roughness (*Ra*) across the three regions of the CFR-PLA material. Each row corresponds to a specific region (Region 1, Region 2, and Region 3), and each column illustrates the pairwise interaction between parameters. The color gradient represents *Ra* values, where darker regions signify smoother surfaces, and lighter regions indicate rougher finishes. Notably, the influence of cutting speed and abrasive feed rate is more pronounced in Region 3, leading to higher variability in *Ra* compared to other regions. These variations underscore the necessity for tailored parameter optimization to achieve consistent surface quality for CFR-PLA. In the first row in Figure 11 (Region 1), *Ra* demonstrates a moderate sensitivity to x1 (pressure) and x3 (abrasive feed rate), with smoother surfaces achievable at lower abrasive feed rates and moderate pressures. This observation aligns with studies reporting that fiber reinforcement in CFR-PLA reduces roughness variability, especially under controlled feed rates [69]. For Region 2, *Ra* appears less sensitive to variations in x1 and x2, indicating a stabilizing effect of fiber reinforcement in this region. However, localized cutting speed optimization and abrasive feed rate could still reduce roughness, suggesting that process control remains important for fine-tuning surface quality [71]. Region 3, in contrast, exhibits higher variability in *Ra* values, mainly influenced by x3. Higher abrasive feed rates increase roughness significantly, which may result from uneven fiber–matrix distribution during processing [70].

The contour plots highlight the need for region-specific optimization, particularly for Region 3, where process parameters have the most pronounced impact on surface roughness. These results align with findings from advanced machining studies that emphasize the role of process control in ensuring uniform surface finishes in fiber-reinforced composites [75,76]. The results underscore the necessity of adjusting processing parameters based on the region-specific behavior of the CFR-PLA to achieve optimal surface quality.

**Figure 12:**
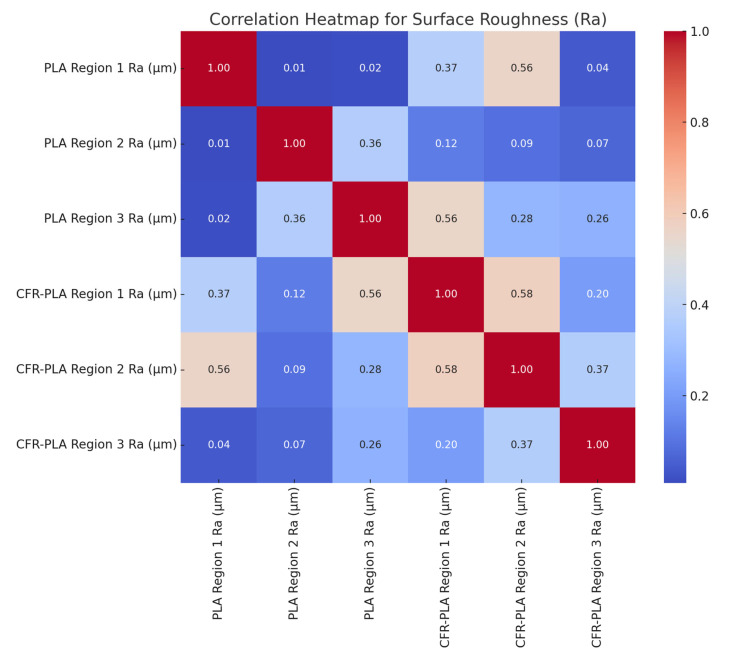
The relationships between surface roughness (*Ra*) values across different regions of PLA and CFR-PLA samples.

The correlation heatmap (Figure 12) illustrates the relationships between *Ra* values across different regions of PLA and CFR-PLA samples, revealing critical insights into inter-regional and cross-material dependencies. For PLA, Region 1 shows minimal correlation with Regions 2 and 3 (~0.01–0.02), indicating independent surface characteristics likely due to localized stabilization during the manufacturing process, where factors such as deposition overlaps and thermal effects have minimal influence in Region 1 [69]. A moderate correlation (0.36) between PLA Regions 2 and 3 suggests shared dependencies on factors such as deposition overlaps and edge effects, which are commonly seen in additive manufacturing, particularly in regions experiencing more direct interaction with the printing surface. In CFR-PLA, stronger correlations are observed, particularly between Region 1 and Region 3 (0.56), highlighting the role of fiber reinforcement in creating consistent surface characteristics across regions. This reflects how carbon fiber reinforcement mitigates surface irregularities, producing a more uniform surface quality [70]. Cross-material correlations, such as the moderate link between PLA Region 3 and CFR-PLA Region 1 (0.56), suggest that specific process parameters influence both materials similarly, emphasizing the stabilizing effect of reinforcement fibers in enhancing dimensional stability across different material compositions [75,76]. These findings underscore the importance of region-specific and material-specific optimization strategies to improve surface quality in additive manufacturing processes, ensuring uniformity and precision in both PLA and CFR-PLA composites.

**Figure 13:**
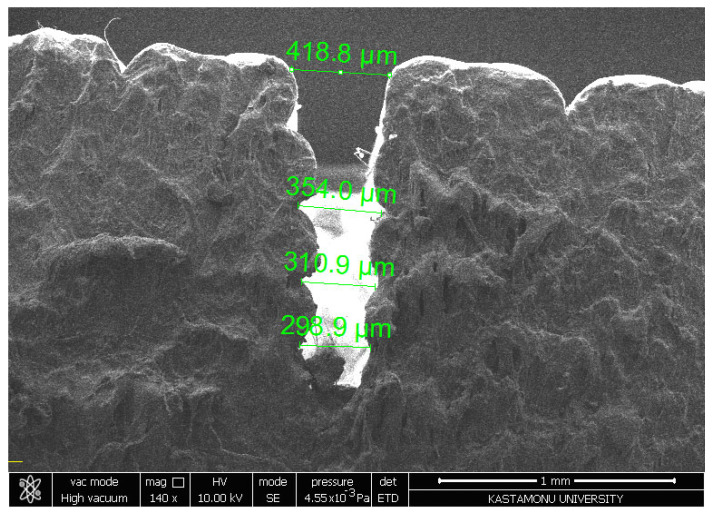
SEM image of PLA samples’ cutting of kerf formation surface.

The SEM image (Figure 13) at 140× magnification reveals the cross-sectional morphology of a machined kerf, displaying tapering from 418.8 µm at the top to 298.9 µm at the bottom, a characteristic effect of abrasive water jet machining (AWJM). The rough and fibrous sidewalls indicate material removal through erosion and brittle fracturing, likely influenced by the anisotropic properties of the composite material, which appears to contain reinforcing fibers. The observed taper and irregular surface texture suggest suboptimal machining parameters, such as abrasive flow rate, traverse speed, or water pressure. Despite the taper, the absence of significant delamination or fiber pull-out highlights the integrity of fiber–matrix bonding. To enhance dimensional accuracy and surface quality, further optimization of AWJM parameters and post-processing techniques is recommended. These observations underscore the importance of tailoring machining conditions to the material’s properties for improved performance in precision applications.

**Figure 14:**
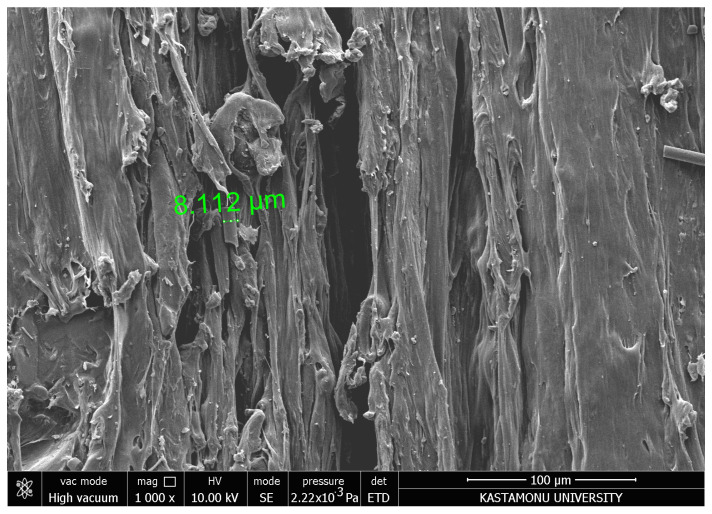
SEM image of CFR-PLA samples’ abrasive particles impacting the carbon fiber filler.

The SEM image (Figure 14) at 1000× magnification reveals detailed surface features with distinct grooves and sharp edges, indicative of the material removal mechanism during abrasive water jet machining (AWJM). The measured groove depth of 8.112 µm highlights localized erosion effects caused by high-pressure abrasive particles impacting the surface. The elongated and irregular surface morphology suggests anisotropic material properties and uneven energy distribution during machining. These features may also result from inadequate optimization of machining parameters, such as traverse speed or abrasive particle flow rate. The lack of significant fiber pull-out or delamination suggests strong matrix–reinforcement bonding, although further refinement of AWJM settings could improve surface smoothness and reduce defects. This analysis emphasizes the importance of precise parameter control to enhance the surface integrity of machined composites.

The optimization values given in Table 9 highlight the impact of AWJM parameters on *Ra* across different regions of PLA and CFR-PLA samples. The optimal water pressure (x1) for both materials was consistently set to 2500 bar, suggesting that moderate pressure levels are adequate to achieve minimal surface roughness. Cutting speed (x2) and abrasive flow rate (x3) varied across regions, indicating that tailored parameter adjustments are necessary depending on the specific region of the material. Region 1 exhibited the lowest *Ra* values for both PLA and CFR-PLA (9 μm), demonstrating a high degree of uniformity and reduced sensitivity to machining conditions. However, Region 3 consistently showed higher *Ra* values, with PLA reaching 15.2 μm and CFR-PLA achieving a lower but notable 12.3 μm. This suggests that edge effects and the interactions between fibers and the matrix in this region require additional optimization to improve surface quality. Overall, CFR-PLA outperformed PLA in all areas, showcasing the stabilizing effect of carbon fiber reinforcement on surface quality during AWJM. These results underline the importance of region-specific optimization and further validate CFR-PLA’s suitability for high-precision applications that demand consistent surface integrity and quality. The findings emphasize that optimizing AWJM parameters based on specific regions and material characteristics can significantly enhance the surface finish, particularly in advanced composite materials like CFR-PLA.

The findings of this study demonstrate that AWJM-processed CFR-PLA offers significant advantages for industrial applications, particularly in the aerospace and automotive sectors. Its reduced taper angle and superior surface finish make it ideal for lightweight structural components, aerodynamic body parts, and functional prototypes where precision and dimensional stability are critical. Additionally, AWJM’s ability to minimize thermal damage and tool wear enhances its suitability for machining intricate geometries and complex designs, ensuring cost-effective and efficient production. The results underscore CFR-PLA’s potential for high-performance applications, where mechanical strength and surface integrity are essential. Future research should build upon these findings by conducting comparative analyses between AWJM and other advanced machining techniques, such as laser cutting and CNC milling, to identify the most effective methods for processing CFR-PLA composites. Exploring hybrid fiber reinforcements and refining post-processing techniques could further improve CFR-PLA’s mechanical and thermal properties, expanding its applicability in fields requiring high precision and sustainability. Additionally, investigating the impact of post-processing methods on surface finish and structural integrity could unlock new potential for CFR-PLA in high-precision engineering applications, where environmental sustainability and superior performance are paramount.

## 4. Conclusions

This study underscores the potential of carbon fiber-reinforced PLA (CFR-PLA) composites as a superior material for precision applications requiring high dimensional accuracy and enhanced surface quality. Through a systematic evaluation of abrasive water jet machining (AWJM) parameters—namely water pressure, cutting speed, and abrasive flow rate—key factors influencing surface roughness and kerf taper angle were identified. The findings revealed that CFR-PLA outperformed pure PLA in terms of surface integrity, with carbon fiber reinforcement significantly minimizing surface irregularities during high-pressure cutting. These results highlight CFR-PLA’s suitability for aerospace and automotive applications, particularly for lightweight structural components requiring tight tolerances.

- Enhanced cutting quality with CFR-PLA: CFR-PLA demonstrated superior performance with reduced kerf taper angles and lower surface roughness compared to PLA, attributed to the stabilizing effect of carbon fibers that enhances dimensional accuracy and mechanical stability during machining.
- Significant influence of AWJM parameters: Water jet pressure and abrasive flow rate were identified as the most critical parameters. High water pressure (3500 bar) and moderate abrasive flow rates (250–450 g/min) consistently improved surface quality and minimized kerf taper angles.
- Impact of traverse speed: Higher traverse speeds (above 1200 mm/min) negatively affected surface roughness and kerf taper angles by reducing jet–material interaction time. Controlled traverse speeds (400–800 mm/min) yielded optimal results.
- Region-specific variability: Surface roughness varied across different regions, influenced by edge effects and fiber–matrix interactions, emphasizing the need for region-specific optimization of AWJM parameters.
- Statistical validation and optimization: ANOVA confirmed the significant impact of AWJM parameters on surface quality. Optimized conditions for both PLA and CFR-PLA were identified, providing a roadmap for achieving high cutting precision.

These findings position CFR-PLA as a promising material for lightweight structural components, functional prototypes, and customized parts in the aerospace and automotive sectors. Its machinability and environmental benefits make it a key candidate for industries emphasizing sustainability and performance. Future research should focus on advanced post-processing techniques, hybrid reinforcement strategies, and comparisons with alternative machining technologies to further enhance CFR-PLA’s utility in precision engineering.

## Figures and Tables

**Table 1:** FDM printing parameters and their values.

Parameter	Value
Layer height	0.2 mm
Layer width	0.4 mm
Wall thickness	0.8 mm
Printing temperature	220 °C
Bed temperature	60 °C
Printing speed	60 mm/s
Displacement speed	100 mm/s
Flow	100%
Raster angle	−45°/45°
Infill density	100%

**Table 2:** Device accuracy and error margin table.

Device	Accuracy Limit	Error Margin
Mitutoyo SJ-210 Surface Roughness Tester	±0.003 mm	±1%
Siemens CNC-Controlled AWJM System	±0.03 mm	±2%
OPTIKA B-510MET Microscope	±1 μm	±5%
KMT Pressure Sensor	±1 bar	±0.5%
Abrasive Flow Rate Control Unit	±5 g/min	±1%

**Table 3:** Parameters and levels determined for cutting test.

Parameters	Codes	Level 1	Level 2	Level 3	Units
Water pressure	x1	2500	-	3500	Bar
Nozzle feed rate	x2	400	800	1200	mm/min
Abrasive flow rate	x3	100	250	450	g/min

**Table 4:** Experimental results.

Exp. No.	Water Pressure (bar)	Nozzle Feed Rate (mm/min)	Abrasive Flow Rate (g/min)
1	3500	400	100
2	3500	400	250
3	3500	400	450
4	3500	800	100
5	3500	800	250
6	3500	800	450
7	3500	1200	100
8	3500	1200	250
9	3500	1200	450
10	2500	400	100
11	2500	400	250
12	2500	400	450
13	2500	800	100
14	2500	800	250
15	2500	800	450
16	2500	1200	100
17	2500	1200	250
18	2500	1200	450

**Table 5:** Summary of cutting taper angles and surface roughness measurements for CFR-PLA samples under various AWJM conditions.

No.	x1 (bar)	x2 (mm/min)	x3 (g/min)	T (°)	Region 1 (μm)	Region 2 (μm)	Region 3 (μm)
1	3500	400	100	1.608	9.186	9.096	12.336
2	3500	400	250	1.338	13.326	10.126	10.136
3	3500	400	450	1.918	9.486	9.216	13.046
4	3500	800	100	2.368	9.826	11.926	17.246
5	3500	800	250	1.778	13.336	9.806	15.056
6	3500	800	450	1.978	14.306	10.986	11.816
7	3500	1200	100	2.518	13.366	10.316	19.376
8	3500	1200	250	2.088	15.406	12.446	30.766
9	3500	1200	450	2.358	15.266	10.926	14.376
10	2500	400	100	2.638	11.336	8.866	10.636
11	2500	400	250	2.308	15.366	11.156	12.886
12	2500	400	450	2.808	13.176	8.546	17.876
13	2500	800	100	3.048	12.996	11.096	16.256
14	2500	800	250	2.178	22.426	12.016	13.986
15	2500	800	450	3.178	16.516	11.776	13.986
16	2500	1200	100	3.088	16.416	11.736	26.336
17	2500	1200	250	2.708	16.796	14.806	16.016
18	2500	1200	450	3.118	13.796	11.736	17.366

**Table 6:** Summary of cutting taper angles and surface roughness measurements for pure PLA samples under various AWJM conditions.

No.	x1 (bar)	x2 (mm/min)	x3 (g/min)	T (°)	Region 1 (μm)	Region 2 (μm)	Region 3 (μm)
1	3500	400	100	3.65	9.476	17.716	20.966
2	3500	400	250	1.54	9.876	8.866	18.646
3	3500	400	450	2.19	10.016	9.296	15.596
4	3500	800	100	2.25	15.546	10.826	18.226
5	3500	800	250	1.77	9.916	10.006	27.066
6	3500	800	450	2.16	12.876	13.066	27.016
7	3500	1200	100	2.35	11.436	17.286	21.786
8	3500	1200	250	1.83	14.646	13.756	24.766
9	3500	1200	450	2.55	20.266	14.196	29.786
10	2500	400	100	2.91	11.996	17.636	24.146
11	2500	400	250	2.21	21.166	10.776	18.386
12	2500	400	450	2.72	15.006	11.086	14.746
13	2500	800	100	3.07	18.046	12.676	18.486
14	2500	800	250	3.18	15.616	14.236	28.516
15	2500	800	450	3.11	22.166	15.126	28.596
16	2500	1200	100	3.06	11.816	13.526	34.626
17	2500	1200	250	2.69	21.126	15.266	20.636
18	2500	1200	450	3.46	27.726	11.896	21.676

**Table 7:** ANOVA for the CFR-PLA.

Source	DF	Adj SS	Adj MS	F-Value	*p*-Value
x1 (bar)	1	1.973	1.9734	0.07	0.794
x2 (mm/min)	2	163.735	81.8677	2.95	0.091
x3 (g/min)	2	0.06	0.03	0	0.999
Error	12	333.587	27.7989		
Total	17	499.355			

**Table 8:** ANOVA for the pure PLA.

Source	DF	Adj SS	Adj MS	F-Value	*p*-Value
x1 (bar)	1	0.079	0.079	0	0.952
x2 (mm/min)	2	203.218	101.609	4.94	0.027
x3 (g/min)	2	17.063	8.532	0.41	0.67
Error	12	246.38	20.578		
Total	17	467.298			

**Table 9:** The optimization table for *Ra* across PLA and CFR-PLA regions.

Region	Optimal x1 (bar)	Optimal x2 (mm/min)	Optimal x3 (g/min)	Lowest *Ra* (μm)
PLA Region 1	2500	600	200	9.0
PLA Region 2	2500	700	250	9.1
PLA Region 3	2500	800	300	15.2
CFR-PLA Region 1	2500	600	200	9.0
CFR-PLA Region 2	2500	700	250	9.1
CFR-PLA Region 3	2500	800	300	12.3

## Data Availability

The data presented in this study are available on request from the corresponding author due to privacy reason.
